# Addressing Digital Disparities in Alzheimer Disease by Improving Access to Alzheimer Resources for Spanish-Speaking Latino or Latina Individuals in Los Angeles County: Mixed Methods Study

**DOI:** 10.2196/67147

**Published:** 2025-08-13

**Authors:** Stephanie Ovalle-Eliseo, Aisha Mohammed, Gabriela Islas Huerta, Keith Vossel, Lorena H Monserratt, Mirella Díaz-Santos

**Affiliations:** 1 Mary S Easton Center for Alzheimer's Research and Care Department of Neurology University of California, Los Angeles Los Angeles, CA United States

**Keywords:** dementia, digital divide, Latino and Hispanic individuals, structural determinants of health, web accessibility

## Abstract

**Background:**

The COVID-19 pandemic disrupted traditional health care delivery models, exacerbating disparities between those with and without ready access to digital technology. This digital divide poses a structural barrier to accessing equitable healthy aging resources and dementia care. Latino and Hispanic individuals, who constitute nearly half of Los Angeles County’s population and face a projected tripling of Alzheimer disease and related dementia (ADRD) prevalence by 2040, are particularly impacted.

**Objective:**

This paper aims to examine the barriers and facilitators affecting access to digital health education and resources for Alzheimer disease (AD) prevention and care management during the COVID-19 pandemic. This study focuses on the digital barriers possibly hindering Spanish-speaking Latino and Hispanic individuals in Los Angeles County from using online services offering critical AD prevention and care resources amid the COVID-19 pandemic.

**Methods:**

We developed a conceptual model based on users’ digital access or web literacy and language as barriers and facilitators impacting access to digital AD prevention and care resources. Between January 2022 and February 2022, we identified 15 websites of local organizations providing digital AD prevention services and resources in Los Angeles County during the pandemic. We applied our digital divide model to qualitatively evaluate the 15 websites. A post hoc analysis was conducted to reevaluate the 15 websites in 2025, and interrater reliability was evaluated using a Cohen κ analysis.

**Results:**

Out of the 15 websites, 5 featured web navigation accessibility tools (4/15 in 2025), 4 provided content available in Spanish (6/15 in 2025), and 2 included resources for family dialogue about AD care and management (3/15 in 2025). One website showed cultural and linguistic responsiveness in its content (2/15 in 2025). Cohen κ analysis revealed substantial agreement for digital acceptability factors including Spanish language (κ=0.71), resources available in Spanish (κ=0.71), and family dialogue resources (κ=0.74). Agreement for web accessibility tools was moderate at (κ=0.53). We uncovered other unforeseen structural barriers to digital access, including email subscription requirements, English language–centered online forms, and the limited availability of Spanish-speaking staff.

**Conclusions:**

Our study highlights structural barriers hindering access to digital AD prevention and care resources tailored to the needs and values of Latino and Hispanic communities living in Los Angeles County. The findings emphasize the need to bridge the digital gap by incorporating user-friendly features and culturally and linguistically responsive elements in website design and implementation. This approach will move our field toward equitable access to digital ADRD prevention and care resources by mitigating structural barriers that sustain ADRD disparities in Latino and Hispanic communities.

## Introduction

### Alzheimer Disease and Its Impact on Latino and Hispanic Individuals

Alzheimer disease (AD) poses an increasing threat to Latino and Hispanic individuals in the United States, who face a 1.5 times higher risk of developing AD [1]. Despite robust efforts, the current treatment options for AD are limited [2-4], necessitating a shift toward preventive measures. This shift involves advocating for healthy aging and increasing brain health awareness within the Latino and Hispanic communities through effective education on prevention, early detection, and care management [5]. Previous literature indicates that a fundamental understanding of a health condition significantly enhances self-efficacy, empowering individuals to make profound behavioral changes that alleviate negative attitudes toward seeking preventive health services [6-8]. For the Latino and Hispanic communities, effective prevention, early identification, and care management are shaped by having a comprehensive network of interventions spanning community connections, including families, friends, and community or faith-based organizations, alongside broader societal, pharmacological, and policy measures [9].

### Health Disparities Amid the Digital Transition During the COVID-19 Pandemic

Before the COVID-19 pandemic, accessible care services and resources, such as educational workshops and caregiving support groups, were available in person [10]. However, with the many political (ie, expanded Medicaid coverage for remote health services) and cultural shifts brought on during the COVID-19 pandemic, the widespread use of eHealth grew exponentially [11-13]. This affected the modality in which health services were being rendered, not just in terms of treatment and care management, but also in accessing education and resources [14]. In the AD field, websites of organizations detailing available AD services and resources to local communities serve as valuable platforms offering preventive health information and dementia care management tools [15-17]. However, the sudden shift to digital platforms exposed stark disparities in access to informational health services, highlighting the digital divide [18,19]. The digital divide refers to the gap between those with adequate access to information via technology and those with limited to no access to technology due to various systemic barriers [20]. The transition to mostly digital resources was particularly challenging for communities disproportionately affected by the pandemic due to policies impacting the equitable distribution of eHealth services, thus widening the existing digital divide [21-23].

### Impact of the Widened Digital Divide on Health Disparities

Despite earnest efforts, engaging with Latino and Hispanic communities via digital platforms has posed significant challenges for many health sectors due to a range of barriers [24,25]. The widened digital divide has further compounded these challenges, as evidenced by lower recruitment and engagement of Latino and Hispanic individuals in informational sessions and events on AD during the COVID-19 pandemic, despite increased promotional efforts [26]. Notably, even with recruitment initiatives led by Spanish bilingual staff, attendees were predominantly English-dominant bilingual and college-educated individuals [26,27]. This discrepancy underscores the impact of barriers, such as limited technology access and low digital literacy, which hindered participants from effectively engaging online.

Digital literacy, defined as the ability to access, evaluate, understand, and use information and communication technologies effectively, plays a pivotal role in addressing these challenges [28]. It encompasses skills, such as navigating digital platforms, critically evaluating online content, and communicating and collaborating with digital or virtual communities. In the context of engaging with Latino and Hispanic communities via digital platforms, digital literacy is essential for individuals to access online health resources, participate in virtual events, and engage in digital communication effectively. However, barriers, such as limited access to technology, low levels of digital literacy, and culturally and linguistically incongruent online content (ie, messaging) can impede individuals’ ability to fully benefit from digital health initiatives, exacerbating existing disparities in health outcomes and access to care [18].

### Socioeconomic, Environmental, and Familial Factors Driving the Digital Divide Among Latino and Hispanic Individuals in Los Angeles

Digital disparities faced by the Latino and Hispanic communities predate the sudden surge in digital reliance caused by the pandemic. A 2016 study showed that 41% of immigrant Latino and Hispanic individuals below the national median income level had internet access only via a mobile device, compared with 17% of US-born counterparts [13,29]. Latino and Hispanic immigrants with lower education levels, lower English proficiency, and lower socioeconomic status were less likely to seek eHealth information due to lower digital literacy [30,31]. Despite the rise in mobile internet usage among Latino and Hispanic individuals with higher education and income [29], individuals with lower incomes faced difficulties with internet connectivity, providing evidence for the existence of the digital divide [32]. A recent Pew Research Center blog on The Internet and Pandemic found that Latino and Hispanic individuals comprised a significant portion of the 30% of adults reporting lower *tech readiness* [32]. People with lower tech readiness lacked confidence in using electronic devices for online tasks and were less likely to deem the internet essential during the pandemic.

The prevalence of health information–seeking tendencies among Latino and Hispanic individuals could be attributed to social determinants of health (SDOH), including barriers beyond internet access [32]. While some groups with low-hazard occupations smoothly transitioned to remote work, Latino and Hispanic individuals, contrarily, found themselves on the front lines as essential workers, taking on the brunt of the pandemic’s physical and emotional toll [33]. This reality, coupled with rising worries regarding insecurities in food, housing, and employment, further complicated the digital transition [34,35]. With the presence of telehealth outlasting the pandemic lockdowns and the widened digital divide gap, it is essential to identify modifiable digital accessibility factors tailored to the needs and preferences of the Latino and Hispanic communities, especially when it comes to digital health education and resources for AD prevention and care management. Recent work has demonstrated that digital engagement and internet use are associated with improved cognition, knowledge of AD, and AD-related prevention behaviors [5,36]. Thus, more effort is needed to increase accessibility for those most vulnerable to the digital divide.

### Objective

The objective of this paper is to further explore the barriers and facilitators that influenced access to digital brain health education and resources for AD prevention and care management during the COVID-19 pandemic. Specifically, this study focuses on website usage barriers that may have contributed to the digital divide in Spanish-speaking Latino and Hispanic communities living in Los Angeles County [26]. Our study serves as a case study in improving digital health information accessibility for Latino and Hispanic individuals, but the model and methods presented can be applied to diverse underrepresented communities. By assessing how local organizations in Los Angeles provide AD-related resources to their community online (48% Latino and Hispanic; 38% Spanish Speaking), we aim to identify pathways for increasing accessibility, ensuring that digital health tools are not only available but also understandable, usable, and relevant for a Latino and Hispanic population in increasing need for these resources [37].

## Methods

### Methodological Framework Guiding Our Review of Digital Health Disparities

**Figure 1:**
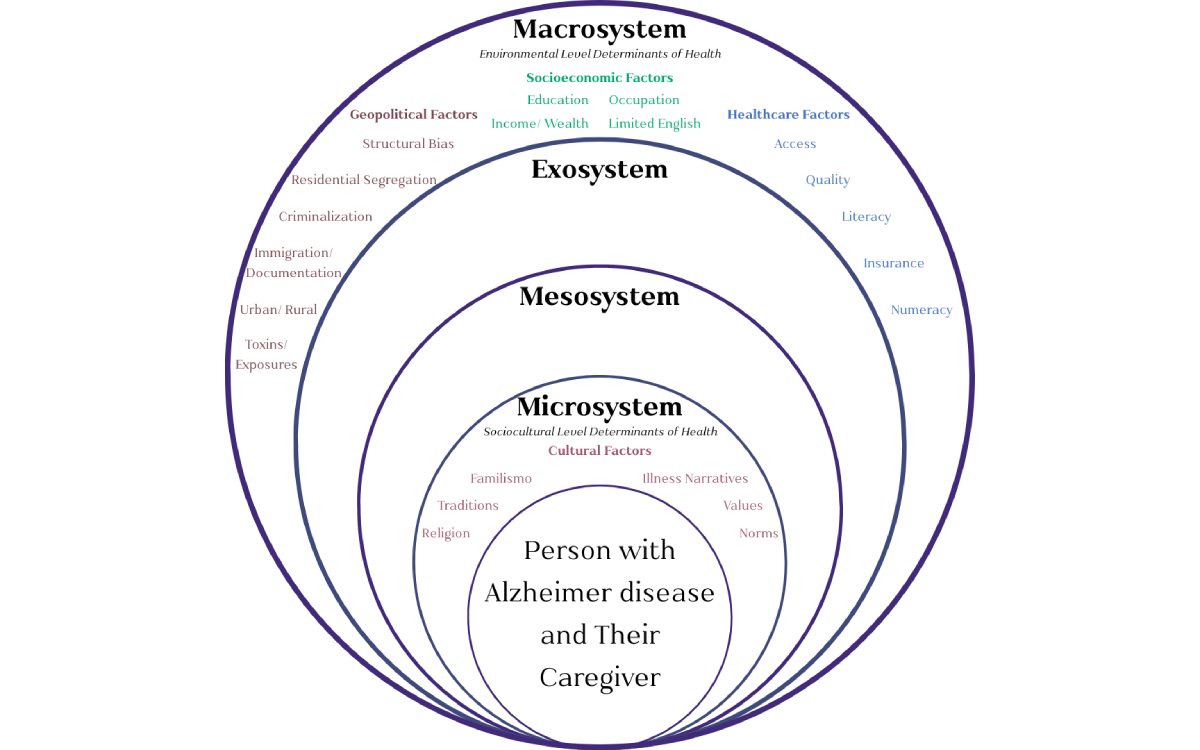
Adapted Bronfenbrenner model of environmental and sociocultural determinants influencing Latino and Hispanic Alzheimer disease (AD) care engagement. An adapted model, based on Bronfenbrenner’s social ecological model [34,67], highlights the environmental factors impacting Latino and Hispanic patients with AD and their caregivers in Los Angeles County. Key elements include familismo and illness narratives, which influence family-centered decision-making by incorporating traditions, religion, values, and norms, consistent with sociocultural determinants of health outlined by the National Institutes on Aging (NIA) [38].

We used the National Institutes on Aging health disparities research framework [38] and adapted Bronfenbrenner’s social ecological model [34] to guide our work. Our conceptual model (Figure 1 [34,38,39]) highlights the possible SDOH at the environmental and sociocultural levels that impact digital literacy among Latino and Hispanic patients with AD and their caregivers living in Los Angeles County.

At the environmental level, determinants, such as geographic and political factors—including documentation status and the criminalization of immigrants—play a significant role in shaping health care accessibility [40]. For example, undocumented Latino and Hispanic individuals may be reluctant to seek health care due to fears of deportation or legal repercussions, which can prevent them from accessing digital resources or even learning about available AD prevention and care management services. Socioeconomic factors such as income and occupation also contribute, as lower-income families may lack the financial resources to afford internet access or digital devices, further widening the digital divide. In addition, health care factors, such as access to insurance and the availability of Latino and Hispanic medical providers, can either facilitate or hinder the ability to obtain and use digital health resources effectively [41,42].

At the sociocultural level, *familismo* [43] and illness narratives [44] play a vital role in family-centered decision-making dynamics, which include traditions, religion, values, and norms that are part of the National Institutes on Aging’s framework for sociocultural determinants of health [38]. In Latino and Hispanic communities, for instance, illness narratives often use terms such as *loquera* (craziness) to describe mental illness, including dementia. This language not only reflects cultural perceptions but also ties into the broader issue of structural stigma against people with mental or cognitive disorders. Such stigma can create barriers to care, perpetuate negative stereotypes, and reinforce social inequities. The use of terms such as *loquera* can contribute to this stigma, framing mental health and dementia conditions in a dismissive way that may discourage individuals and families from seeking support or accessing appropriate care [45]. Combined, these environmental and sociocultural factors potentially contributed to the uptake of digital health information and help-seeking behaviors for eHealth AD prevention and care management resources and services during the COVID-19 pandemic.

### Digital Literacy Model Development

**Figure 2:**
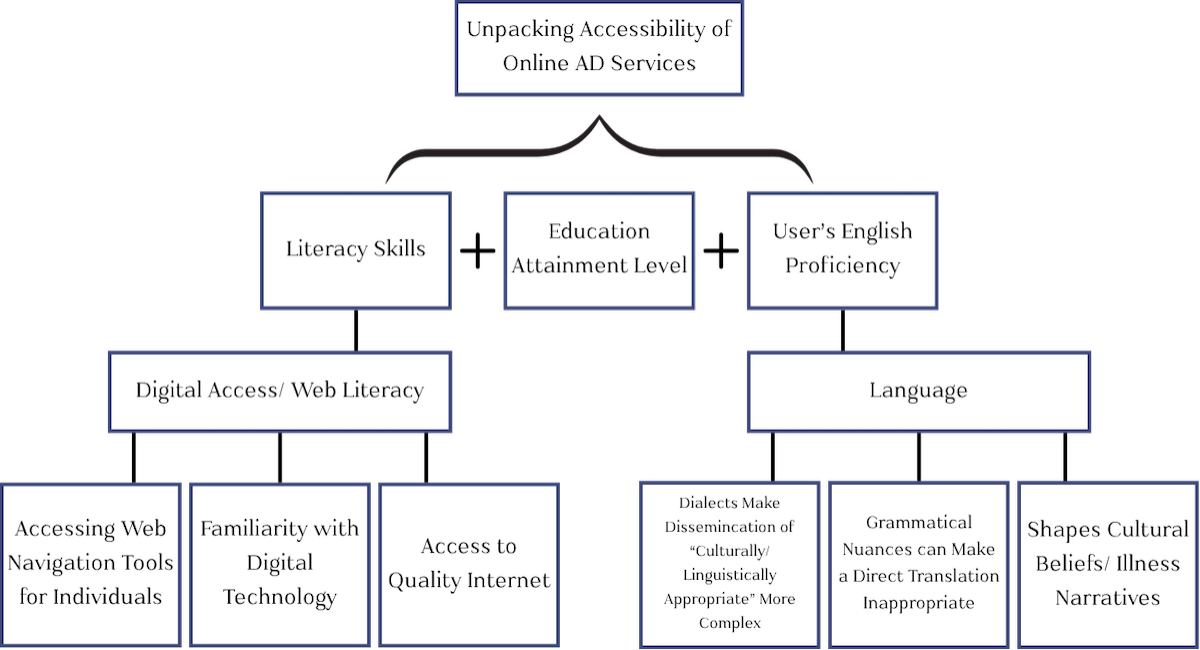
The digital literacy model for understanding online Alzheimer disease (AD) resource accessibility. A bivariate conceptual model hypothesized to conceptually link the relationship between a user’s digital reach (digital access and web literacy) and the nuanced complexities of virtually accommodating for preferred language as modifiable factors that can facilitate accessibility of online AD resources and services.

Using UCLA Library Search, which provides access to multiple external databases, we conducted a literature review of studies from January 2000 to February 2022 that assessed the role of the digital divide in driving health accessibility disparities among racial and ethnic minorities. Specifically, we accessed databases, such as PubMed, Google Scholar, and MEDLINE, to identify repeated digital accessibility barriers and facilitators. Search keywords included COVID-19, digital divide, eHealth, online health information, digital access, Latinos, Hispanics, marginalized communities, and health equity. This search identified nearly 40 relevant articles in which access to quality internet, familiarity with technology use, disabilities and cognitive impairment, educational attainment, age, and language [13,26,30,31,46-49] were the main factors elucidated as contributing to a web user’s overall digital literacy, which is the ability to find, evaluate, and engage with online health information [28]. Through this review, we recognized the absence of a comprehensive model that conceptualizes these themes in relation to digital literacy and its role in accessing online AD prevention and care management services and resources. To address this gap, we developed the digital literacy model to help us investigate why Latino and Hispanic individuals in Los Angeles County may not frequently engage with AD services and resources online (Figure 2).

The development of this model was an iterative process guided by insights from the literature review and enhanced by our research team’s lived experiences as members of the Latino and Hispanic communities, in addition to collaborating with these communities. Over the course of 2 months, the first author (SOE) and senior author (MDS) engaged in frequent and regular working sessions, mapping out and discussing the implications of the repeated themes identified in the literature. Initial drafts took the form of hand-drawn diagrams, which were refined through ongoing discussions and digitalized into the final model. The fourth author (KV; director of the Mary S. Easton Center for Alzheimer’s Research & Care at the time) reviewed the final model.

Our bivariate model conceptually links the relationship between a user’s digital reach (digital access and web literacy) and preferred language as modifiable factors that can facilitate accessibility of online AD services and resources. Furthermore, it also helps refine a focus on environmental determinants of health at the geographic, political, and social levels [38]. It identifies two primary factors—(1) *digital access or web literacy* and (2) *language*—to better understand potential drivers influencing a user’s experience in accessing online AD services and resources. *Digital access or web literacy* are contingent on a user’s need or preference for *accessible web navigation tools*, their *familiarity with digital technology*, and their *access to quality internet*. Similarly, a user’s web navigation experience is shaped by their *preferred language* and the depth to which the complexities of its written dissemination are embedded and considered. That is, dialects make dissemination of *culturally and linguistically appropriate* content more complex, grammatical nuances can make a direct translation inappropriate; and language itself shapes cultural beliefs or illness narratives. Altogether, the extent to which the two primary factors will shape the outcome of a user’s web navigation experience is dependent on the individual’s literacy skills, education attainment levels, and user’s English proficiency. We implemented several factors of our conceptual model when evaluating websites that offer AD prevention and care management resources and services for Los Angeles County’s constituents, 38% of whom speak Spanish [37].

Notably, the initial research team was composed of 2 Spanish and English-bilingual (Spanish as first language) Latina women with backgrounds in research and engagement with underrepresented communities. We acknowledge that our lived experiences influenced the way we approached both the literature review and the conceptualization of this model. Our familiarity with barriers and facilitators to digital access and health literacy within marginalized communities informed the way we identified and interpreted key themes in the literature. This perspective also shaped our understanding of how existing digital health frameworks may not fully capture the cultural and structural factors affecting engagement with web-based AD services among Latino and Hispanic populations. Our individual and collective intersectionality and positionality as bilingual and bicultural researchers influenced the development of our digital literacy model, such that language accessibility was treated as a core determinant of digital engagement rather than a secondary factor [50-52]. By incorporating this lens into our model, we sought to highlight the specific digital barriers faced by these communities and propose actionable insights for improving accessibility and inclusivity in web-based AD resources.

### Digital Search Process

Between January 2022 and February 2022, we conducted a Google search using a combination of words (including the Spanish counterpart) and terms to explore community organizations that offer AD prevention, early detection, and care management resources and services in Los Angeles County. Key words included were as follows: *Alzheimer’s and Related Dementias* (*Alzheimer y Demencias Relacionadas*), *Alzheimer’s* (*Alzheimer*), *dementia(s)* (*demencia/s*), *elder adult services* (*servicios para adultos mayores*), *healthy aging* (*envejecimiento sano*), *aging services* (*servicios de envejecimiento*), *social services* (*servicios sociales*), *human services* (*recursos humanos*), *seniors* (*ancianos*), *community resources* (*recursos comunitarios*), *Los Angeles*, *Los Angeles County* (*condado de Los Ángeles*). To ensure a comprehensive search, we conducted searches separately in English and Spanish rather than using combined searches. For example, a search for “Healthy aging services in Los Angeles County” was followed by “Servicios para el envejecimiento sano en el Condado de Los Ángeles” to account for language variations in how AD-related services might be described. We identified 89 organizations with associated digital websites.

### Website Screening and Selection Criteria

**Figure 3:**
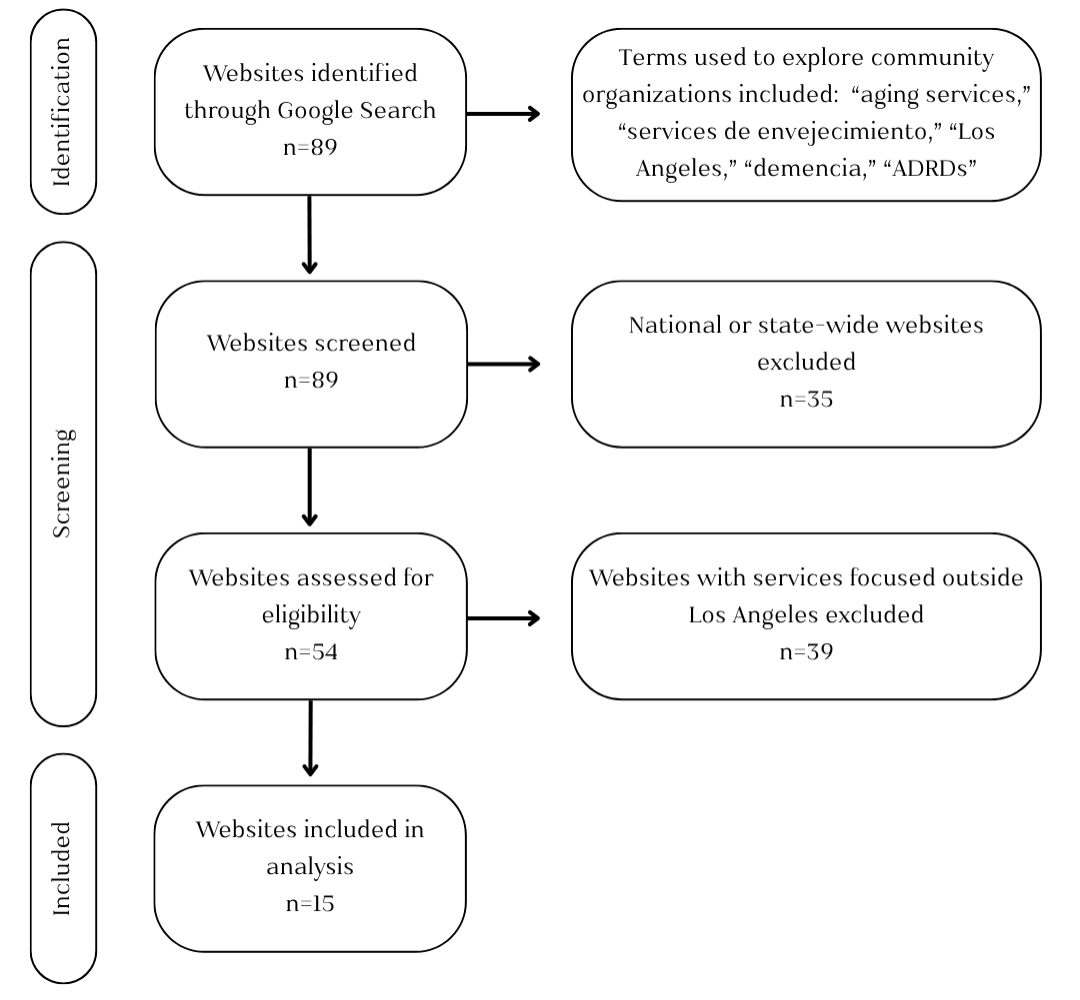
Flowchart of the selection process for the qualitative review of websites’ content. ADRD: Alzheimer disease and related dementia.

Our study’s objective was to assess the digital accessibility of websites offering AD prevention and care management resources and services in Los Angeles County. To ensure a systematic selection process, the first author (SOE) conducted the initial website screening under the supervision and guidance of the senior author (MDS). Throughout the process, they met regularly to discuss observations and interpret findings in relation to how the conceptual model helped identify potential digital navigation barriers and facilitators on each website. This iterative approach refined our screening criteria and strengthened the evaluation of website accessibility factors. While the senior author (MDS) did not complete an independent search, she had knowledge of the community-based organizations in Los Angeles County providing aging and AD-related services because of her outreach collaborations before COVID. Websites were included if they represented locally established organizations providing AD prevention and case management educational information through in-person community events or webinars, caregiving support (eg, adult day care, family resources, referral services, support groups, social services support, or meal support), or resources for professionals, with all information available on the official website. Of 89 resulting websites in the initial search, 15 entities met these criteria. Figure 3 outlines our selection screening process for the qualitative review of websites’ accessibility factors with our model.

The first screening step (Websites Screened) involved reviewing website titles and home page descriptions to determine whether an entity was a local organization or a state or federally run agency. We excluded 21 state-level organizations identified in the initial search as they were not within Los Angeles County and provided services beyond direct healthy aging or AD-related services. For instance, 4 focused on planning healthy aging improvement task forces, such as the California Task Force on Alzheimer’s Prevention & Preparedness and the California Master Plan on Aging. While they are crucial state-led initiatives, they offered no immediate aid to families navigating an AD prevention and case management. An additional 14 national-level entities were also excluded, as their resources and services were not exclusively tailored to constituents of Los Angeles County. This group included the National Institute on Aging and the National Hospice and Palliative Care Organization, 2 other federally funded education and research organizations, 4 caregiver aide service locators, and 6 financial education and training support organizations for caregivers. A total of 35 California state and nationwide organizations were excluded as they did not offer specific support services outlined in the inclusion criteria. A total of 54 websites were then reviewed in greater detail (assessed for eligibility) to determine whether their services were specifically available to families in Los Angeles County. If an organization offered AD services, such as respite care or caregiver support, but only served families outside of Los Angeles County (eg, Orange County), the website was excluded from the final analysis. In this step, 39 were excluded for providing services related to our inclusion criteria outside of Los Angeles County. Following this two-step screening and eligibility process, 15 websites met our final inclusion criteria and key digital accessibility factors were analyzed.

### Evaluation Process

#### Overview

We assessed the 15 websites following our digital literacy model (Figure 2). All 15 websites were evaluated by the following key digital accessibility factors: (1) web accessibility tools, (2) the type and number of services or resources available in Spanish (in addition to English), (3) the inclusion of linguistic and cultural barriers, and (4) the inclusion of family dialogue resources. We chose these 4 key digital accessibility factors because they represent fundamental components that influence how Latino and Hispanic communities can access and engage with online AD-related information. Although our model encompasses various elements of digital literacy, these 4 factors were selected for assessment due to their critical role in shaping the user experience, particularly for populations facing systemic barriers. By evaluating the consistency of these factors across websites, we aimed to identify patterns in website design that may either facilitate or hinder equitable access to digital health resources and services for Spanish and English speakers in the Latino and Hispanic communities.

The first author (SOE) systematically assessed each website for these criteria, while the senior author (MDS) supervised the process and provided guidance to ensure a consistent approach across website evaluations. Throughout the review, they met regularly to discuss findings and their implications in relation to the conceptual model, allowing a structured and iterative evaluation process.

#### Web Accessibility Tools

To assess the presence of sufficient web accessibility tools, our team accounted for the inclusion of user-friendly navigation and adjustment tools, such as screen reader compatibility, available color adjustment tools, text adjustment tools, and other measures aimed at improving web accessibility to those protected under the American Disability Act [53]. Past research suggests such features can help ensure that Latino and Hispanic individuals in Los Angeles with cognitive impairments, often comorbid with other disabilities, can access vital information with reasonable accommodations [54].

#### Service Availability in Spanish

The type and number of services or resources available in Spanish (in addition to those offered in English) were defined as critical indicators of linguistic inclusivity and cultural responsiveness within the scope of this study. For example, for every resource listed on a particular webpage (eg, written informational discourse or a listed caregiver support group), we asked, “Was there a Spanish version available?” If the answer was yes, we also assessed whether Google Translate software was implemented to increase language accessibility, whether other translation tools were used, or whether the information existed on a linked page exclusively written in Spanish.

#### Linguistic and Cultural Barriers: Illness Depiction and Word Choices

The first and senior author’s own lived experiences and backgrounds in underrepresented communities shaped how we approached this digital accessibility factor. Notably, the senior author’s clinical expertise is in cultural neuropsychology with Spanish monolingual and Spanish or English bilingual older adults, those with possible AD, and caregivers. Our familiarity with Spanish dialects from diverse Latino and Hispanic regions played a critical role in identifying potential linguistic barriers and facilitators that may not be immediately evident to those outside these communities. This positionality allowed us to evaluate websites through a culturally competent lens, particularly in assessing whether illness narratives, caregiving frameworks, and resource availability were aligned with the realities of Spanish-speaking families navigating AD. We leveraged the research team’s familiarity with colloquial Spanish from Mexico, Central America, South America, and Latin America to evaluate potential linguistic and cultural barriers embedded in the written discourse of online health information, recognizing that words and the ways they are conveyed carry specific cultural influences, particularly within Latino and Hispanic communities. We delved into the concept of illness narratives, which play a significant role in shaping and molding the experience of illness for individuals and their families [44]. Understanding the nuances of these narratives is crucial, especially in the context of AD, where describing the disease’s prognosis implications and offering support must be approached in a culturally informed and competent manner [9,55]. For example, as we explored the websites’ content, we assessed word choices used to depict AD: *enfermedad* versus *mal* (“sickness” vs “bad condition”) or *demencia* versus *discapacidad cognitiva* (“dementia” vs “cognitive impairment”).

#### Family Dialogue Facilitation Resources

We also assessed for availability of family dialogue or support services or resources. The role of *familismo,* family-centered decision-making and multigenerational roles in Latino and Hispanic households [43,55], is a valuable tool to assist families and extended communities in preventing, detecting, and navigating the AD care systems.

#### Verification via Direct Inquiry

In addition to the online assessment, we conducted direct inquiries by calling 6 organizations identified on the websites. We chose 6 of the 15 organizations due to an existing partnership or expressed interest in forming one. These calls aimed to further assess the status of the programs listed on the websites. Specifically, we inquired about the currency of the information, seeking confirmation that the programs and services were still active and relevant. Moreover, we sought insights into the organizations’ plans for future programming, especially those originally available only in English, to determine if there were considerations for making these resources accessible in Spanish. This phone-based inquiry allowed us to gather real-time information and insights directly from the organizations, providing a comprehensive understanding of the current and potential linguistic inclusivity of the resources offered.

This part of the study was deemed exempt from Human Subjects Research under 45 CFR 46, as it involved gathering publicly available information about organizational resources rather than identifiable private data.

### Data Analysis

We systematically analyzed each website for the presence or absence (yes or no) of the 4 digital acceptability factors aligned with our digital literacy model, including web accessibility tools feature, type and number of services or resources available in Spanish, linguistic or cultural barriers (illness depiction and word choices), and family dialogue facilitation resources. These variables were identified as critical determinants of a website’s accessibility and usability based on our research team’s positionality and the guiding framework of the digital literacy model. Five research questions (RQs) were asked per site to assess accessibility.

1. Is there a web accessibility tool featured on the site’s home page? Is this embedded into the website (eg, font adapter) or is it facilitated through a third party entity (eg, UserWay.org [56])?
2. Is the entire site available in Spanish or is information in Spanish found under specific tabs (eg, “En Español”)? Are all services and resources offered in English also available in Spanish?
3. Is the information presented in Spanish conveyed in a culturally congruent language?
4. Are resources available to help facilitate family dialogues about AD, implications for the future, and caregiving?
5. Are there additional barriers in website navigation and access to available eHealth AD resources?

We conducted a qualitative assessment to evaluate the availability and implementation of the 4 digital accessibility factors identified by our digital literacy model across the selected 15 websites. First, the first and senior author identified and defined the 4 accessibility factors, ensuring they were directly aligned with our RQs. The first author then systematically reviewed each website and discussed with the senior author the presence or absence of each factor. Microsoft Excel was used to organize the data, with columns corresponding to each factor and rows representing individual websites. For each website, the first author recorded detailed observations of how each factor was implemented, including any barriers or facilitators identified. Data were organized into structured fields corresponding to the RQs, allowing a transparent evaluation of accessibility and usability patterns.

#### Post Hoc Analysis

A post hoc analysis was conducted on April 23, 2025, to evaluate the current accessibility and availability of resources on the 15 websites approximately 3 years later. No phone inquiries were performed at this time. The second author (AM) conducted this review by answering the initial RQ for each of the websites and met with the senior author to discuss her findings. A Cohen κ analysis was conducted to measure inter-rater and interdate reliability of the responses.

## Results

### Website Accessibility Assessment

Our analyses of web navigation accessibility tools revealed varying levels of support across 15 websites. Five websites incorporated tools designed to enhance user experience, such as font size adjustment, contrast adaptation, and text-to-speech functionality. Notably, 1 of 5 websites used the EqualWeb software, offering additional accessibility features [57]. Among the 15 websites, 4 provided informational content in Spanish. One of the websites demonstrated a comprehensive and culturally nuanced approach to Spanish translation, delivering information in an empathetic tone (eg, “Cuidar a una persona con pérdida de memoria es difícil”*—*caring for a person with memory loss is difficult [58]). The other 3 websites used formal translations with a matter-of-fact tone with two of these websites using Google Translate [53,59]. Examples include, “Aprenda sobre la enfermedad y qué puede esperar*”* (learn about the disease and what you can expect), and “para obtener más información, visite nuestra pestaña Centros Multipropósito para Personas Mayores” (For more information, visit our Multipurpose Senior Centers eyelash [tab]) [59]. The use of the word *pestaña* (“eyelash” in English) as the Spanish translation for “tab” is contextually wrong. Moreover, 9 of 15 websites listed at least one resource or service, such as caregiver support groups, digital workshops, or downloadable tip sheets, as being available in Spanish, despite the written content of the website being available in English only. Only 2 of 15 websites provided resources to facilitate dialogue about AD among family members. Of the 6 organizations contacted, we found that 2 of the organizations, which offer a wide array of services, greeted the first author with a phone tree in English before routing her to the desired respondent. The other 4 greeted the first author with an English-speaking receptionist. One person informed us she also spoke Spanish, whereas the other 3 mentioned their Spanish-speaking skills were limited, but they could understand general questions regarding the services their respective organization provides.

Across the assessment, 3 websites required an email subscription to further access services or information, potentially creating hurdles for community members who are cautious about sharing personal information online. Another barrier to sustained digital engagement, across 5 websites, was the encouragement to submit questions through English online inquiry forms instead of dialing a direct number, thus introducing an extra layer of complexity for users seeking immediate assistance.

### Post Hoc Analysis

The post hoc analysis revealed changes in website accessibility from the initial assessment. These changes include the inclusion or discontinuation of web navigation tools, increased usage of google translate for web translations, increased availability of Spanish resources, and increased availability of family dialogue resources (Table 1). Multimedia Appendix 1 provides a detailed breakdown of each evaluated website, including the date accessed (initial and post hoc), presence of web accessibility tools, availability of Spanish-language content, and inclusion of family dialogue resources.

**Table 1:** Summary of evaluated websites and accessibility features.

Organization name and date accessed		Web accessibility tools present (yes or no)	Written content available in Spanish (yes or no)	At least one listed resource or service available in Spanish (yes or no)	Family dialogue facilitation resources (yes or no)	Additional barriers
**Alzheimer’s Association [60]**						
	January 28, 2022	No	Yes	Yes	Yes	Multiple phone call routing options Limited Spanish-fluent speaking staff by phone call
	April 23, 2025	No	Yes	Yes	Yes	N/A^a^
**Alzheimer’s Los Angeles [58]**						
	January 27, 2022	No	Yes	Yes	Yes	Limited Spanish- fluent speaking staff by phone call
	April 23, 2025	No	Yes	Yes	Yes	Not entirely translated to Spanish. Only certain tabs for caregivers
**City of Los Angeles Department of Aging** **[59]**						
	February 7, 2022	Yes	Yes	Yes	No	Used Google Translate
	April 23, 2025	No	Yes	Yes	No	Used Google Translate
**DMH^b^ Genesis Program [53]**						
	February 7, 2022	Yes	Yes	Yes	No	Used Google Translate
	April 23, 2025	Yes	Yes	Yes	No	Used Google Translate
**Jewish Family Services [61]**						
	February 3, 2022	Yes	No	No	No	Limited Spanish-fluent speaking staff by phone call
	April 23, 2025	Yes	No	No	No	N/A
**LA County Department of Public Health, Health and Aging [62]**						
	February 7, 2022	Yes	No	No	No	N/A
	April 23, 2025	Yes	No	No	No	N/A
**Leeza’s Care Connection [63]**						
	February 4, 2022	No	No	No	No	N/A
	April 23, 2025	No	No	No	No	N/A
**USC^c^ Los Angeles Caregiver Resource Center [64]**						
	February 4, 2022	No	No	Yes	No	Digitally submitted inquiries encouraged
	April 23, 2025	No	No	Yes	No	N/A
**Mary S Easton Center for Alzheimer’s Research and Care [65]**						
	January 27, 2022	No	No	No	No	N/A
	April 23, 2025	No	Yes	Yes	Yes	Used Google Translate Family conversation materials only available in English
**ONE Generation [66]**						
	January 27, 2022	No	No	Yes	No	Digitally submitted inquiries encouraged Email subscription for further access Multiple phone call routing options
	April 23, 2025	No	No	Yes	No	N/A
**Optimistic People in a Caring Atmosphere (OPICA) [67]**						
	February 3, 2022	No	No	Yes	No	Digitally submitted inquiries encouraged
	April 23, 2025	No	No	Yes	No	N/A
**Rancho Los Amigos CADC^d^ [68]**						
	January 28, 2022	No	No	Yes	No	Digitally submitted inquiries encouraged
	April 23, 2025	No	Yes	Yes	No	Used Google Translate
**Saint Barnabas Senior Services [69]**						
	February 2, 2022	No	No	No	No	Email subscription for further access
	April 23, 2025	No	No	Yes	No	N/A
**South Central Los Angeles Emerging Aging Disability Resource Connection [70]**						
	February 3, 2022	Yes	No	No	No	Limited Spanish-fluent speaking staff by phone call
	April 23, 2025	No	No	No	No	N/A
**WISE and Healthy Aging [71]**						
	January 31, 2022	No	No	Yes	No	Email subscription for further access Digitally submitted inquiries encouraged
	April 23, 2025	Yes	No	Yes	No	N/A

^a^N/A: not applicable.

^b^DMH: Department of Mental Health.

^c^USC: University of Southern California.

^d^CADC: California Alzheimer’s Disease Centers of California.

Our Cohen κ analysis revealed substantial agreement for Spanish language (κ=0.71), resources available in Spanish (κ=0.71), and family dialogue resources (κ=0.74). Reliability of the analysis of web accessibility tools was moderate at (κ=0.53). Table 2 presents the results of the inter-rater reliability analysis using Cohen κ.

**Table 2:** Interrater reliability.

Category	Cohen κ^a^ (SE; 95% CI)	*P* value^b^
Web accessibility tools	0.53 (0.25; 0.04-1.02)	.03
Spanish language	0.71 (0.19; 0.33-1.09)	<.001
Resources available in Spanish	0.71 (0.19; 0.33-1.09)	<.001
Family dialogue resources	0.74 (0.20; 0.34-1.14)	<.001

^a^Cohen κ is a measure of interrater reliability κ values are interpreted as follows: κ≤0: no agreement, κ=0.01 to 0.20: slight agreement, κ=0.21 to 0.40: fair agreement, κ=0.41 to 0.60: moderate agreement, κ=0.61 to 0.80: substantial agreement, and κ=0.81 to 1.00: almost perfect agreement.

^b^*P*<.05 is considered statistically significant.

## Discussion

### Principal Findings

In examining the landscape of AD prevention and care management resources accessible to Spanish- and English-speaking Latino and Hispanic individuals living in Los Angeles County, our analysis of 15 websites has unveiled critical insights into the digital disparities faced by these communities. The limited presence of written content in Spanish, with only 4 of the 15 websites providing such content (6/15 in 2025), underscores the pressing need for targeted improvements in language accessibility. Notably, the variation in language adaptation quality among these websites indicates the importance of using a nuanced and empathetic approach in delivering information [55]**,** as observed in the comprehensive use of Spanish by 2 standout websites (Alzheimer’s Association, Alzheimer’s Los Angeles). In addition, we found that only 5 of 15 websites included web accessibility tools (4/15 in 2025) and just 2 provided resources to support family dialogues (3/15 in 2025), making these websites less user-friendly and relevant overall [49,54]. A post hoc website assessment in 2025 revealed relatively high reliability between the 2 raters, with the 3 categories—Spanish language, resources available in Spanish, and family dialogue resources—having substantial reliability between raters. One category, web accessibility tools, showed moderate reliability. Notably, changes in web design between the 2022 and 2025 assessments affected scoring. Some sites either rolled back on or added accessibility tools, some added Spanish language, and some added Spanish resources, highlighting the consistent iteration and revision of websites. Despite these changes, the need for additional accessibility, language availability, and resources persists.

Our study unveiled additional barriers to sustained digital engagement. The requirement for email subscriptions on 3 websites and the preference for online inquiry forms on 5 websites may inadvertently create obstacles for community members who are cautious about sharing personal information or seeking immediate assistance [35,40]. In 2025, we also noted that while a few sites improved Spanish-language interfaces or added new resources, these updates were inconsistently applied across all evaluated sites, meaning some aspects were available in Spanish while others remained English only. In addition, our phone inquiries in 2022 with the community-based organizations brought attention to the limited availability of Spanish proficient and fluent speakers, a concern that significantly impacts effective communication and the understanding of available services.

### Cultural and Linguistic Diversity in Los Angeles

Our study, conducted in the context of the significant and diverse Latino and Hispanic population in Los Angeles County, illuminates critical issues surrounding digital accessibility of AD prevention and care management resources. With nearly half of the county’s population identifying as Latino or Hispanic, comprising a mosaic of cultural backgrounds, our findings emphasize the necessity for targeted initiatives promoting AD-related eHealth educational services and resources tailored to the unique needs of distinct Latino and Hispanic individuals. Latino and Hispanic individuals in Los Angeles County comprise nearly half of the total population, totaling 4.8 million individuals [54]. This ethnic group is marked by cultural and linguistic diversity, with 73% of the Latino and Hispanic population being of Mexican heritage, 9.6% Salvadorian, 6.0% Guatemalan, 3.1% originating from a South American country, 1.3% Honduran, and the remainder from Puerto Rican, Cuban, Dominican, other Central American, or other Hispanic-Latino origins [54]. This rich diversity accentuates the necessity for promoting AD eHealth educational resources and services tailored to these distinct Latino and Hispanic subgroups in a culturally and linguistically competent manner.

### Barriers to Digital Engagement During the Pandemic

In response to the significant shift toward digital platforms during the pandemic, which prompted our investigation into the digital landscape, community members were notably *not* engaging with webinars [26,47]. While we recognize the pandemic’s effect on fundamental necessities within Latino and Hispanic communities, such as access to health care, food, and housing [21,33], our research highlights how the digital realm exacerbates existing disparities. This circumstance led us to develop a digital literacy model designed to assess the website accessibility of organizations providing AD prevention and care management services in Los Angeles County. These websites play a vital role as resources for community members grappling with an AD diagnosis, particularly amid the challenges posed by the pandemic.

### Website Accessibility and Inclusivity

Our analysis revealed multiple barriers to accessing essential AD prevention and care management services through these websites. Specifically, the website designs demonstrated limited consideration for the intricate relationship between a user’s digital competencies (digital access and web literacy) and the nuanced complexities of virtually accommodating for preferred language as modifiable factors that can facilitate accessibility of online AD services. Post hoc assessment showed that despite some language availability improvements in 2025, many websites continued to use translation plug-ins to offer multiple languages. Notably, of the 6 websites available in Spanish in 2025, 4 used Google Translate. The other 2 used culturally adapted language with an empathetic tone but still posed accessibility barriers, as individuals would need to know how to navigate to the right corner to enable the translation. Moreover, the telephone-based inquiry in 2022 suggests that programs and website development in languages other than English (Spanish, in this case) are implemented only as needed [72].

Limited English proficiency has been frequently highlighted as a factor that makes it difficult for users to interact with online health information [24,30]. According to bloggers within the linguistics and translation communities, this is partially due to the lack of adequate translation software accounting for idiom recognition, significant context, and the chronemics or perception of time or timelines across cultures [73]. In other words, Google translations tend to offer more “direct” or literal translations, which do not fit in seamlessly with the delivery of linguistically and culturally accessible AD resources and services to the Latino and Hispanic communities. Furthermore, during our 2025 review, we observed that even when Spanish language resources were provided, there was inconsistent formatting and navigation between English and Spanish when clicking through pages and resources, reducing overall usability. Nonetheless, navigating the website to convert the information into the preferred language with Google Translate often requires some level of English language proficiency and website navigation literacy to access the Spanish interface of a website [47].

Among the 15 websites assessed, only 5 included crucial navigation tools (4/15 in 2025). Notably, our post hoc analysis revealed that 2 websites rolled back their navigation tool, whereas one added web navigation tools. These tools are essential for web and content accessibility, catering to individuals with visual, motor, auditory, speech, or cognitive disabilities, including AD and their caregivers. With nearly 1 in 4 US citizens having one of these disabilities and 46% of people aged 60 years and older being affected, prioritizing accessibility is crucial [74]. Guidelines such as the Web Content Accessibility Guidelines have been established to facilitate the creation of inclusive digital content, recognizing the importance of web navigation tools to enhance accessibility [75]. Approximately one-third of the evaluated sites demonstrated proper attention to fonts, colors, language, navigation structure, and accessibility features, ensuring that Latino and Hispanic individuals in Los Angeles with cognitive impairments can access vital information with reasonable accommodations [76,77].

### Cultural Relevance and Familismo

Incorporating the principle of *familismo* [43,55] into our model, we recognize its pivotal role in enhancing engagement and accessibility to AD resources and services. Our exploration of the role of *familismo* stems from the acknowledgment that involving family members is not only beneficial for seeking AD information and resources but also holds broader applicability across health contexts. Previous research supports this notion, as participants in a study evaluating the acceptability and usage barriers of electronic patient portals in Los Angeles, facilitated by the Department of Health Services, expressed a clear preference for community and family-focused networks of engagement [12]. Their feedback emphasized the significance of familial involvement in efforts to augment knowledge on health topics.

Among the 15 websites assessed, 2 provided resources to support family dialogues (3/15 in 2025). The role of *familismo* in information delivery is highlighted, indicating that involving family and community networks in disseminating AD eHealth information may enhance acceptability and engagement among Latino and Hispanic individuals in Los Angeles. Socioeconomic factors, such as education, income stability, occupation, and English proficiency, along with health care factors such as access, insurance, quality, and health literacy, are crucial SDOH that organizations are encouraged to consider as they develop their website designs and eHealth services and resources [38].

The digital literacy model, with its focus on web accessibility and language considerations, provides a framework for addressing the identified barriers in future website designs. As digital health information persists beyond the COVID-19 era, it is imperative to ensure that such information, particularly resources for AD prevention and care, is made equitable for Latino and Hispanic individuals in Los Angeles County and beyond.

### Limitations and Future Directions

Our study has several limitations. No formal inter-rater reliability assessment was conducted in January to February 2022. While the senior author guided, supervised, and corroborated the key determinants of the model, their definition, and the codebook, the senior author did not complete an independent website review. The second author conducted an independent review of the 15 websites in April 2025 and calculated the Cohen κ coefficient. This postdoc analysis, while still a limitation to the original inter-rater reliability, revealed substantial to moderate agreements between the 2 authors. Variance was largely due to website updates.

Although the post hoc analysis revealed website design changes, we were unable to consistently assess or monitor the changes, making a comprehensive and comparative analysis difficult. To address this, researchers could develop a systematic approach for monitoring changes over time, using web scraping tools or software applications for automated monitoring [78,79]. Regular assessments at predefined intervals would allow for tracking changes in linguistic and literacy levels and identifying emerging trends.

A formal assessment of linguistic and literacy levels in the Spanish content present on the websites was not possible. Future studies should consider collaborating with linguists or language experts specializing in Spanish language and literacy to conduct formal assessments [39,80]. Leveraging standardized tools and methodologies for linguistic and literacy assessment, as well as using software designed specifically for linguistic analysis, can enhance the reliability and validity of findings [72]. In addition, establishing partnerships with academic linguists or language departments at universities can provide valuable insights and guidance on best practices for assessment [81].

Another important consideration is how our research team’s positionality may have shaped our approach to assessing linguistic and cultural barriers in digital health resources. Our positionalities strengthened our ability to identify gaps in culturally responsive digital content; however, we acknowledge that our interpretations may carry implicit biases [82]. Future studies should incorporate diverse research teams with varied backgrounds and expertise to mitigate potential biases and expand perspectives on digital health accessibility.

Despite these limitations, our study serves as a case study of how digital health disparities can be addressed for populations at higher risk, and with higher prevalence, of AD. While our findings might not be broadly generalizable, our framework and approach can be adapted to increase digital health information accessibility for other groups.

Finally, to further enhance digital health accessibility, future studies should explore more in-depth the integration of accessibility software tools, such as screen readers, into websites providing AD-related information. These tools can significantly benefit individuals with disabilities and their families who seek information on AD prevention and care, ensuring that digital platforms are more inclusive and accessible. Investigating the potential impact of such tools on improving usability for people with cognitive and visual impairments would be a key area for research.

Moving forward, we aim to test the validity of the variables in our digital literacy model by incorporating its concepts into the design and implementation of our research laboratory’s own website and targeted digital social media campaign [83-85]. Future research should also explore the SDOH that exist within the mesosystem and exosystem as represented in Bronfenbrenner’s model, which was not covered in this paper. Understanding the interactions between the immediate settings of the mesosystem, such as the relationship between family and health care providers, and the broader social contexts of the exosystem, such as community support networks and policy environments, can offer deeper insights into the systemic factors influencing digital health accessibility for Latino and Hispanic communities.

### Conclusion

In conclusion, this study emphasizes the critical need to bridge the gap between organizations providing AD resources and services and the diverse Latino and Hispanic communities in need of these resources and services living in Los Angeles County. Our study revealed that despite constituting nearly half of the county’s population and increased projected AD prevalence by 2040 [1], Latino and Hispanic individuals are likely to face significant challenges in accessing digital AD information and support services, underscoring existing health disparities in prevention, early detection, and management. By embracing innovative approaches and adapting our model to the evolving digital landscape, our objective is to bridge the information gap, enhance engagement, and contribute to developing culturally and linguistically responsive digital resources for AD prevention and care management.

## Appendix

Multimedia Appendix 1 Summary of evaluated websites and accessibility.

## Abbreviations

AD: Alzheimer disease
RQ: research question
SDOH: social determinants of health

## Data Availability

The datasets generated or analyzed during this study are not publicly available, as we want to ensure the privacy and protection of the organizations we engaged for this project; however, these are available from the corresponding author on reasonable request.

## References

[1] Alzheimer's disease facts and figures. Alzheimer's Association.

[2] Cummings J (2023). Anti-amyloid monoclonal antibodies are transformative treatments that redefine Alzheimer's disease therapeutics. Drugs.

[3] Edwards M, Corkill R (2023). Disease-modifying treatments in Alzheimer's disease. J Neurol.

[4] Self WK, Holtzman DM (2023). Emerging diagnostics and therapeutics for Alzheimer disease. Nat Med.

[5] Neter E, Chachashvili-Bolotin S, Erlich B, Ifrah K (2021). Benefiting from digital use: prospective association of internet use with knowledge and preventive behaviors related to Alzheimer disease in the Israeli survey of aging. JMIR Aging.

[6] Ayalon L, Areán PA (2004). Knowledge of Alzheimer's disease in four ethnic groups of older adults. Int J Geriatr Psychiatry.

[7] Bandura A (2004). Health promotion by social cognitive means. Health Educ Behav.

[8] Dominick GM, Dunsiger SI, Pekmezi DW, Marcus BH (2013). Health literacy predicts change in physical activity self-efficacy among sedentary Latinas. J Immigr Minor Health.

[9] Stroud C, Larson EB, National Academies of Sciences, Engineering, and Medicine, Health and Medicine Division, Board on Health Care Services, Board on Health Sciences Policy, Committee on Care Interventions for Individuals with Dementia and Their Caregivers (2021). Complexity of systems for dementia care, services, and supports. Meeting the Challenge of Caring for Persons Living with Dementia and Their Care Partners and Caregivers: A Way Forward.

[10] Kennedy MA, Hatchell KE, DiMilia PR, Kelly SM, Blunt HB, Bagley PJ, LaMantia MA, Reynolds CF 3rd, Crow RS, Maden TN, Kelly SL, Kihwele JM, Batsis JA (2021). Community health worker interventions for older adults with complex health needs: a systematic review. J Am Geriatr Soc.

[11] Health IT legislation. Assistant Secretary for Technology Policy.

[12] Casillas A, Perez-Aguilar G, Abhat A, Gutierrez G, Olmos-Ochoa TT, Mendez C, Mahajan A, Brown A, Moreno G (2019). Su salud a la mano (your health at hand): patient perceptions about a bilingual patient portal in the Los Angeles safety net. J Am Med Inform Assoc.

[13] Cherewka A (2020). The digital divide hits U.S. immigrant households disproportionately during the COVID-19 pandemic. Migration Policy Institute.

[14] Ramírez-Saltos D, Acosta-Vargas P, Acosta-Vargas G, Santórum M, Carrion-Toro M, Ayala-Chauvin M, Ortiz-Prado E, Maldonado-Garcés V, González-Rodríguez M (2023). Enhancing sustainability through accessible health platforms: a scoping review. Sustainability.

[15] Iribarren S, Stonbraker S, Suero-Tejeda N, Granja M, Luchsinger JA, Mittelman M, Bakken S, Lucero R (2019). Information, communication, and online tool needs of Hispanic family caregivers of individuals with Alzheimer's disease and related dementias. Inform Health Soc Care.

[16] Robillard JM, Feng TL (2017). Health advice in a digital world: quality and content of online information about the prevention of Alzheimer's disease. J Alzheimers Dis.

[17] Duggleby W, Ploeg J, McAiney C, Fisher K, Jovel Ruiz K, Ghosh S, Peacock S, Markle-Reid M, Williams A, Triscott J, Swindle J (2019). A comparison of users and nonusers of a web-based intervention for carers of older persons with Alzheimer disease and related dementias: mixed methods secondary analysis. J Med Internet Res.

[18] Alkureishi MA, Choo ZY, Rahman A, Ho K, Benning-Shorb J, Lenti G, Velázquez Sánchez I, Zhu M, Shah SD, Lee WW (2021). Digitally disconnected: qualitative study of patient perspectives on the digital divide and potential solutions. JMIR Hum Factors.

[19] Chang BL, Bakken S, Brown SS, Houston TK, Kreps GL, Kukafka R, Safran C, Stavri PZ (2004). Bridging the digital divide: reaching vulnerable populations. J Am Med Inform Assoc.

[20] Lythreatis S, Singh SK, El-Kassar AN (2022). The digital divide: a review and future research agenda. Technol Forecast Soc Change.

[21] Gwynn RC (2021). Health inequity and the unfair impact of the COVID-19 pandemic on essential workers. Am J Public Health.

[22] Guiding an improved dementia experience (GUIDE) model. Centers for Medicare & Medicaid Services.

[23] Tousi B (2020). Dementia care in the time of COVID-19 pandemic. J Alzheimers Dis.

[24] De Jesus M, Xiao C (2012). Predicting internet use as a source of health information: a “language divide” among the Hispanic population in the United States. Policy Internet.

[25] Gelman CR (2010). Learning from recruitment challenges: barriers to diagnosis, treatment, and research participation for Latinos with symptoms of Alzheimer's disease. J Gerontol Soc Work.

[26] Gutiérrez Á, Cain R, Diaz N, Aranda MP (2022). The digital divide exacerbates disparities in Latinx recruitment for Alzheimer's disease and related dementias online education during COVID-19. Gerontol Geriatr Med.

[27] Liang J, Aranda MP (2023). The use of telehealth among people living with dementia-caregiver dyads during the COVID-19 pandemic: scoping review. J Med Internet Res.

[28] Digital literacy. American Library Association Literacy Clearinghouse.

[29] Brown A, López G, Lopez MH (2016). Digital divide narrows for Latinos as more Spanish speakers and immigrants go online. Pew Research Center.

[30] Gonzalez M, Sanders-Jackson A, Emory J (2016). Online health information-seeking behavior and confidence in filling out online forms among Latinos: a cross-sectional analysis of the California Health Interview Survey, 2011-2012. J Med Internet Res.

[31] Din HN, McDaniels-Davidson C, Nodora J, Madanat H (2019). Profiles of a health information-seeking population and the current digital divide: cross-sectional analysis of the 2015-2016 California health interview survey. J Med Internet Res.

[32] McClain C, Vogels EA, Perrin A, Sechopoulos S, Rainie L (2021). Navigating technological challenges. Pew Research Center.

[33] Martinez LE, Bustamante A, Balderas-Medina Anaya Y, Domínguez-Villegas R, Santizo-Greendwood S, Diaz S, Hayes-Bautista D (2020). COVID-19 in vulnerable communities: an examination by race/ethnicity in Los Angeles and New York City. UCLA Latino Policy & Politics Initiative.

[34] Bronfenbrenner U (1979). The Ecology of Human Development: Experiments by Nature and Design.

[35] Salgado de Snyder VN, McDaniel M, Padilla AM, Parra-Medina D (2021). Impact of COVID-19 on Latinos: a social determinants of health model and scoping review of the literature. Hisp J Behav Sci.

[36] Kamin ST, Lang FR (2020). Internet use and cognitive functioning in late adulthood: longitudinal findings from the survey of health, ageing and retirement in Europe (SHARE). J Gerontol B Psychol Sci Soc Sci.

[37] The Spanish language in Los Angeles. Los Angeles Almanac.

[38] Hill CV, Pérez-Stable EJ, Anderson NA, Bernard MA (2015). The national institute on aging health disparities research framework. Ethn Dis.

[39] Casanovas-Marsal JO, Civitani Monzón E, Ferrer Duce MP, González de la Cuesta D, Yelmo Valverde R, Pérez Repiso V, Goicoechea Manterola I, de Arriba Muñoz A (2024). Study protocol of translation into Spanish and cross-cultural adaptation and validation of the Problem Areas in Diabetes-Pediatric version (PAID-Peds) survey. Nurs Open.

[40] Adkins-Jackson PB, George KM, Besser LM, Hyun J, Lamar M, Hill-Jarrett TG, Bubu OM, Flatt JD, Heyn PC, Cicero EC, Zarina Kraal A, Pushpalata Zanwar P, Peterson R, Kim B, Turner RW 2nd, Viswanathan J, Kulick ER, Zuelsdorff M, Stites SD, Arce Rentería M, Tsoy E, Seblova D, Ng TK, Manly JJ, Babulal G (2023). The structural and social determinants of Alzheimer's disease related dementias. Alzheimers Dement.

[41] Martínez LE, Anaya Y, Santizo Greenwood S, Diaz SF, Wohlmuth CT, Hayes-Bautista DE (2022). The Latino resident physician shortage: a challenge and opportunity for equity, diversity, and inclusion. Acad Med.

[42] Balderas-Medina Anaya Y, Hsu P, Martínez LE, Hernandez S, Hayes-Bautista DE (2022). Latina women in the U.S. physician workforce: opportunities in the pursuit of health equity. Acad Med.

[43] Del Río N (2010). The influence of Latino ethnocultural factors on decision making at the end of life: withholding and withdrawing artificial nutrition and hydration. J Soc Work End Life Palliat Care.

[44] Kleinman A (1988). The Illness Narratives: Suffering, Healing, and the Human Condition.

[45] Brewer KB, Gibson R, Tomar N, Washburn M, Giraldo-Santiago N, Hostos-Torres LR, Gearing RE (2024). Why culture and context matters: examining differences in mental health stigma and social distance between Latino individuals in the United States and Mexico. J Immigr Minor Health.

[46] Gonzalez M, Sanders-Jackson A, Wright T (2019). Web-based health information technology: access among Latinos varies by subgroup affiliation. J Med Internet Res.

[47] Casillas A, Abhat A, Vassar SD, Huang DY, Mahajan AP, Simmons S, Lyles C, Portz J, Moreno G, Brown AF (2021). Not speaking the same language-lower portal use for limited English proficient patients in the Los Angeles safety net. J Health Care Poor Underserved.

[48] Yoon H, Jang Y, Vaughan PW, Garcia M (2020). Older adults' internet use for health information: digital divide by race/ethnicity and socioeconomic status. J Appl Gerontol.

[49] Mitchell UA, Chebli PG, Ruggiero L, Muramatsu N (2019). The digital divide in health-related technology use: the significance of race/ethnicity. Gerontologist.

[50] Muhammad M, Wallerstein N, Sussman AL, Avila M, Belone L, Duran B (2015). Reflections on researcher identity and power: the impact of positionality on community based participatory research (CBPR) processes and outcomes. Crit Sociol (Eugene).

[51] Secules S, McCall C, Mejia JA, Beebe C, Masters AS, L. Sánchez‐Peña M, Svyantek M (2021). Positionality practices and dimensions of impact on equity research: a collaborative inquiry and call to the community. J Eng Educ.

[52] Jamieson MK, Govaart GH, Pownall M (2023). Reflexivity in quantitative research: a rationale and beginner's guide. Soc Personal Psychol Compass.

[53] GENESIS - Geriatric Services Intervention Support Programs. Los Angeles County Department of Mental Health.

[54] Hispanics/Latinos in Los Angeles county by the numbers. Los Angeles Almanac.

[55] Flores G, Abreu M, Schwartz I, Hill M (2000). The importance of language and culture in pediatric care: case studies from the Latino community. J Pediatr.

[56] UserWay.

[57] EqualWeb.

[58] Alzheimer's Los Angeles.

[59] City of Los Angeles Department of Aging.

[60] California Southland Chapter. Alzheimer's Association.

[61] Jewish Family Service LA.

[62] Health and aging services. County of Los Angeles Public Health.

[63] Leeza's Care Connection.

[64] LA CRC - resources for family caregivers in Los Angeles County. Los Angeles Caregiver Resource Center.

[65] UCLA, Alzheimer's, dementia, memory clinic, doctors, physicians. The Mary S. Easton Center for Alzheimer’s Research and Care at UCLA.

[66] ONEgeneration.

[67] OPICA.

[68] Rancho Los Amigos.

[69] St. Barnabas Senior Services.

[70] Communities Actively Living Independent & Free.

[71] Wise and Healthy Aging.

[72] Ozorio Dutra SV, Chee V, Clochesy JM (2023). Adapting an educational software internationally: cultural and linguistical adaptation. Educ Sci.

[73] (2022). Why isn’t Google translate enough for your global business?. LinkedIn.

[74] Krupa A, Roark JB, Barrett KB (2022). The critical role of web accessibility in health information access, understanding, and use. American Health Information Management Association Foundation.

[75] Alajarmeh N (2022). Evaluating the accessibility of public health websites: an exploratory cross-country study. Univers Access Inf Soc.

[76] Americans with Disabilities Act of 1990, as amended. U.S Department of Justice Civil Rights Division.

[77] (2021). Making your website senior friendly: tips from the National Institute on Aging and the National Library of Medicine. National Institute on Aging.

[78] Kempny C, Brzoska P (2023). [Web scraping applications in health services research: for web experts only, or a tool for every health services researcher?!]. Z Evid Fortbild Qual Gesundhwes.

[79] Speckmann F (2021). Web scraping: a useful tool to broaden and extend psychological research. Zeitschrift für Psychologie.

[80] Montero-Errasquín B, Vaquero-Pinto N, Sánchez-Cadenas V, Geerinck A, Sánchez-García E, Mateos-Nozal J, Ribera-Casado JM, Cruz-Jentoft AJ (2022). Spanish translation, cultural adaptation and validation of the SarQoL®: a specific health-related quality of life questionnaire for sarcopenia. BMC Musculoskelet Disord.

[81] Khalilizadeh Ganjalikhani M, Hesabi A, Ketabi S (2023). Evaluating linguistic comprehensibility of Persian healthcare translations in multilingual contexts: a case study of health translations website from the Victorian Government of Australia. Int J Multiling.

[82] Galdas P (2017). Revisiting bias in qualitative research: reflections on its relationship with funding and impact. Int J Qual Methods.

[83] Equity for Latinx-Hispanic Healthy Aging Lab.

[84] ELHA UCLA. Instagram.

[85] The equity for Latinx-Hispanic healthy aging lab at UCLA. Facebook.

