# Supplementary material for: Addressing Digital Disparities in Alzheimer Disease by Improving Access to Alzheimer Resources for Spanish-Speaking Latino or Latina Individuals in Los Angeles County: Mixed Methods Study
Source: J Med Internet Res. 2025 Aug 13;27:e67147. doi: 10.2196/67147 (PMC12391846; doi:10.2196/67147)
Supplement: Multimedia Appendix 1 [file jmir_v27i1e67147_app1.docx]

**Table 1: Summary of Evaluated Websites and Accessibility Features**

This table provides a detailed breakdown of each evaluated website, including the date accessed, presence of web accessibility tools, availability of Spanish-language content, and inclusion of family dialogue resources.

| Organization’s Name | Date Accessed | Web Accessibility Tools Present (Yes/No) | Written Content Available in Spanish (Yes/No) | At Least One Listed Resource or Service Available in Spanish | Family Dialogue Facilitation Resources (Yes/No) | Additional Barriers |
| --- | --- | --- | --- | --- | --- | --- |
| Alzheimer's Association [62] | 01/28/22 | No | Yes | Yes | Yes | - Multiple Phone Call Routing Options  - Limited Spanish-Fluent Speaking Staff by Phone Call |
| Alzheimer's Los Angeles [63] | 01/27/22 | No | Yes | Yes | Yes | - Limited Spanish- fluent Speaking Staff by Phone Call |
| City of Los Ángeles Dept of Aging [64] | 02/07/22 | Yes | Yes | Yes | No | - Used Google Translate |
| DMH Genesis Program [65] | 02/07/22 | Yes | Yes | Yes | No | - Used Google Translate |
| Jewish Family Services [66] | 02/03/22 | Yes | No | No | No | - Limited Spanish- fluent Speaking Staff by Phone Call |
| LA County Dept of PH, Health and Aging [67] | 02/07/22 | Yes | No | No | No | n/a |
| Leeza's Care Connection [68] | 02/04/22 | No | No | No | No | n/a |
| USC Los Angeles Caregiver Resource Center [69] | 02/04/22 | No | No | Yes | No | - Digitally Submitted Inquiries Encouraged |
| Mary S. Easton Center for Alzheimer's Research and Care [70] | 01/27/22 | No | No | No |  | n/a |
| ONE Generation [71] | 01/27/22 | No | No | Yes | No | - Digitally Submitted Inquiries Encouraged  - Email Subscription for Further Access  - Multiple Phone Call Routing Options |
| OPICA [72] | 02/03/22 | No | No | Yes | No | - Digitally Submitted Inquiries Encouraged |
| Rancho Los Amigos CADC [73] | 01/28/22 | No | No | Yes | No | - Digitally Submitted Inquiries Encouraged |
| Saint Barnabas Senior Services [74] | 02/04/22 | No | No | No | No | - Email Subscription for Further Access |
| South Central Los Ángeles Emerging Aging Disability Resource Connection (SCLA ADRC) [75] | 02/03/22 | Yes | No | No | No | - Limited Spanish- fluent Speaking Staff by Phone Call |
| WISE and Healthy Aging [76] | 01/31/22 | No | No | Yes | No | - Email Subscription for Further Access  - Digitally Submitted Inquiries Encouraged |
| Total Number | 15 | 5 | 4 | 9 | 2 | 12 |
